# Supplementary figures and images for: Fecal bacteria transplantation replicates aerobic exercise to reshape the gut microbiota in mice to inhibit high-fat diet-induced atherosclerosis
Source: PLoS One. 2025 Feb 4;20(2):e0314698. doi: 10.1371/journal.pone.0314698 (PMC11793757; doi:10.1371/journal.pone.0314698)

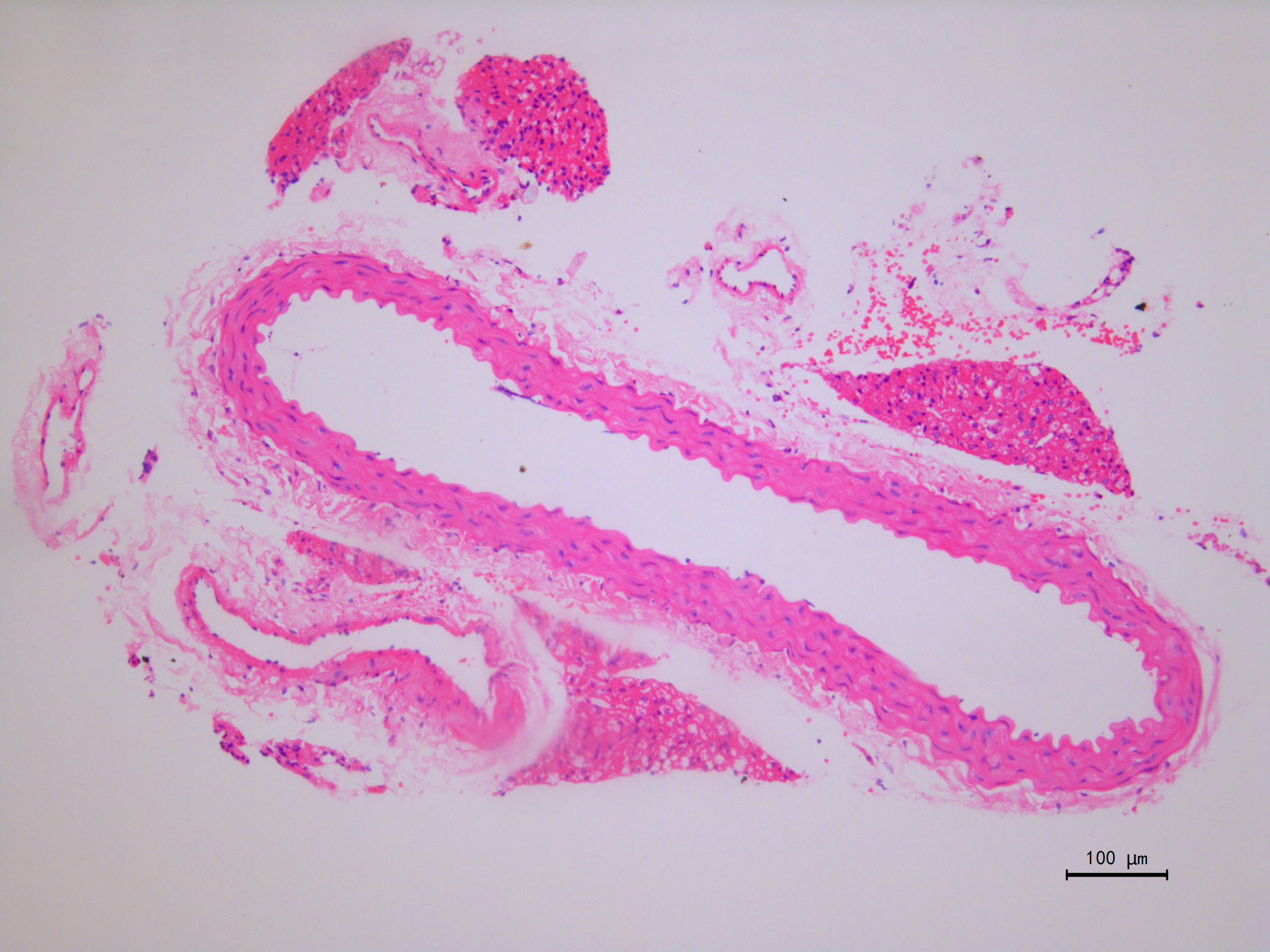

Supplement: S1 Fig — (TIF) [file pone.0314698.s002.tif]

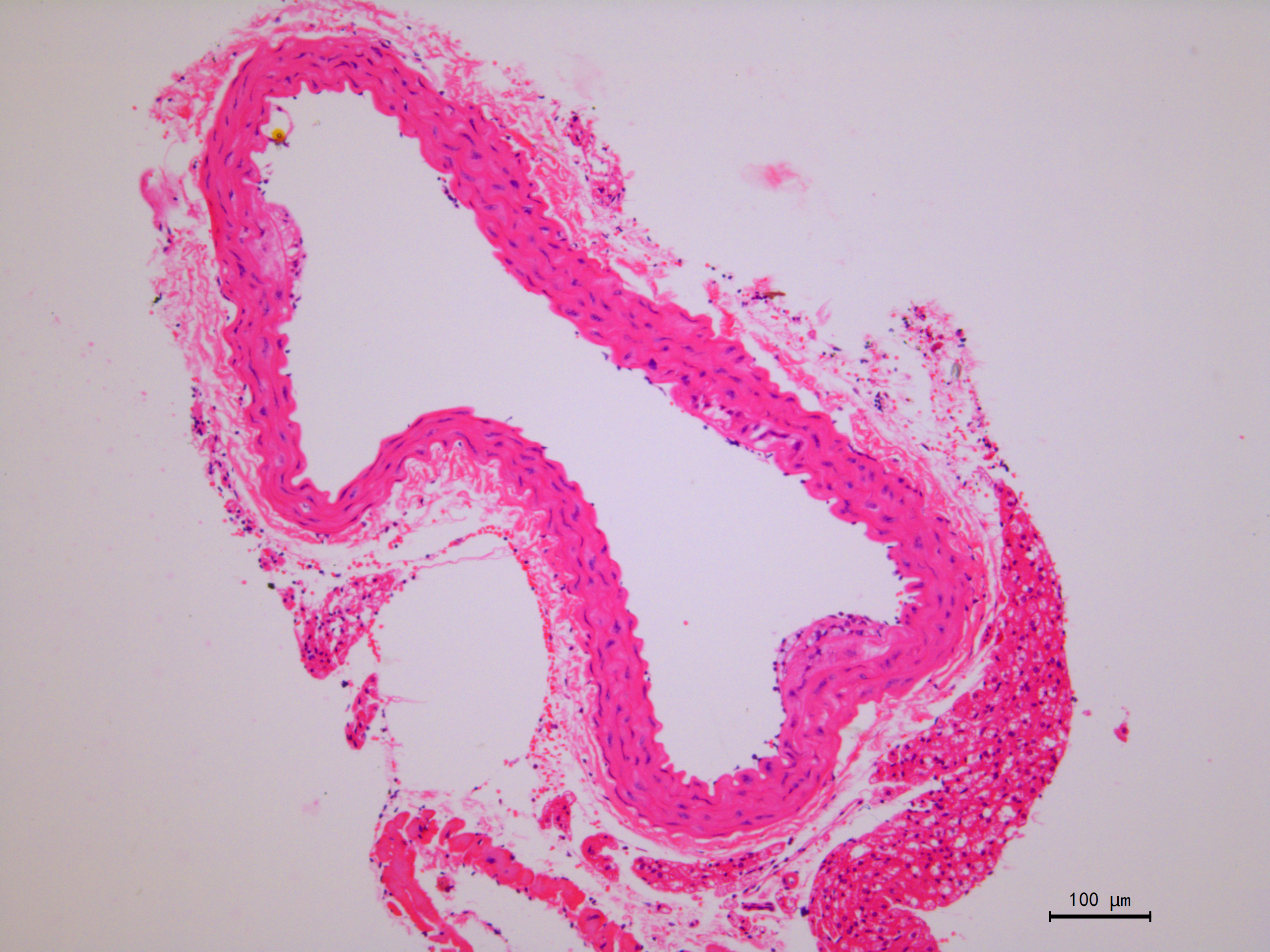

Supplement: S2 Fig — (TIF) [file pone.0314698.s003.tif]

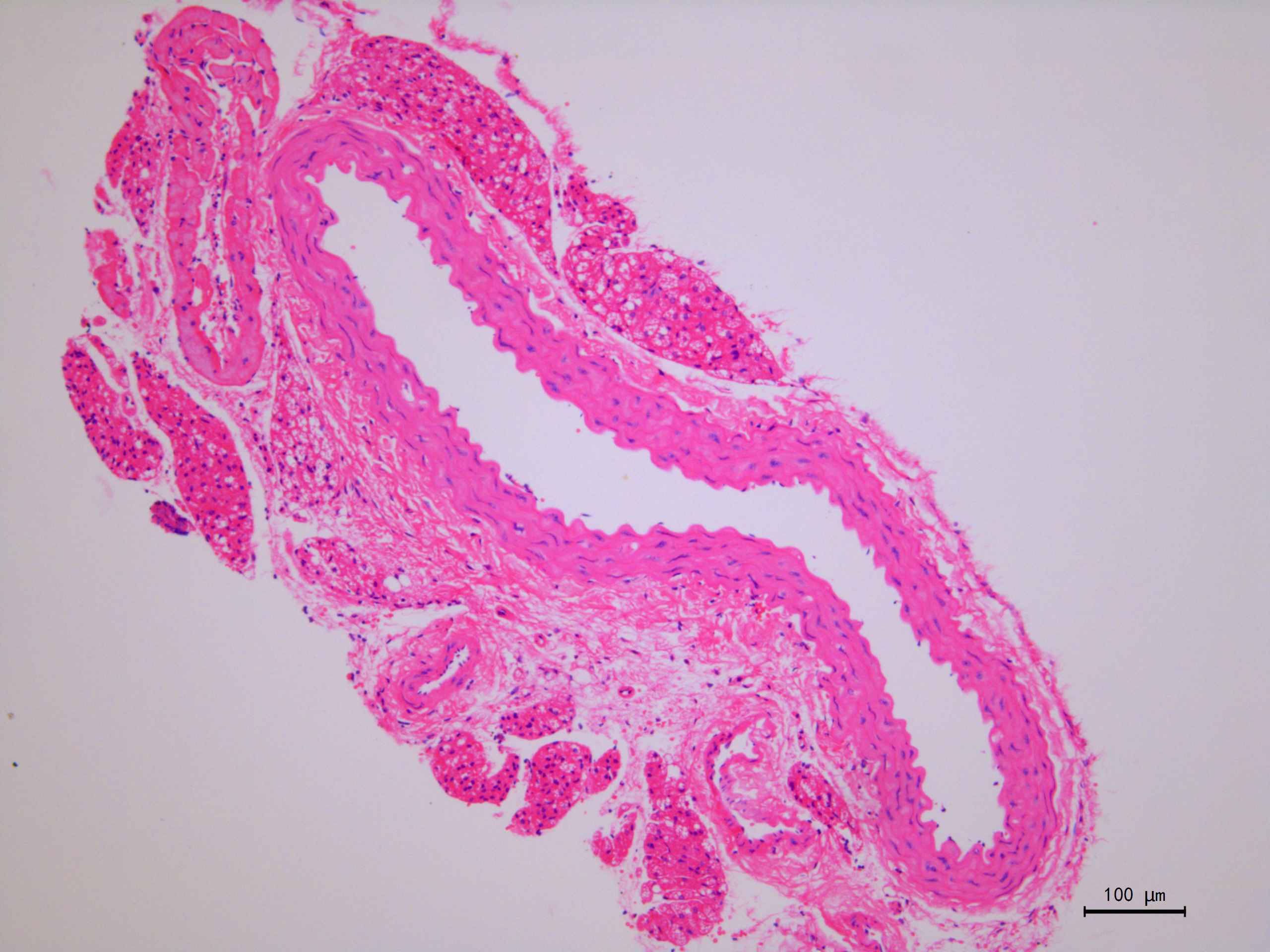

Supplement: S3 Fig — (TIF) [file pone.0314698.s004.tif]

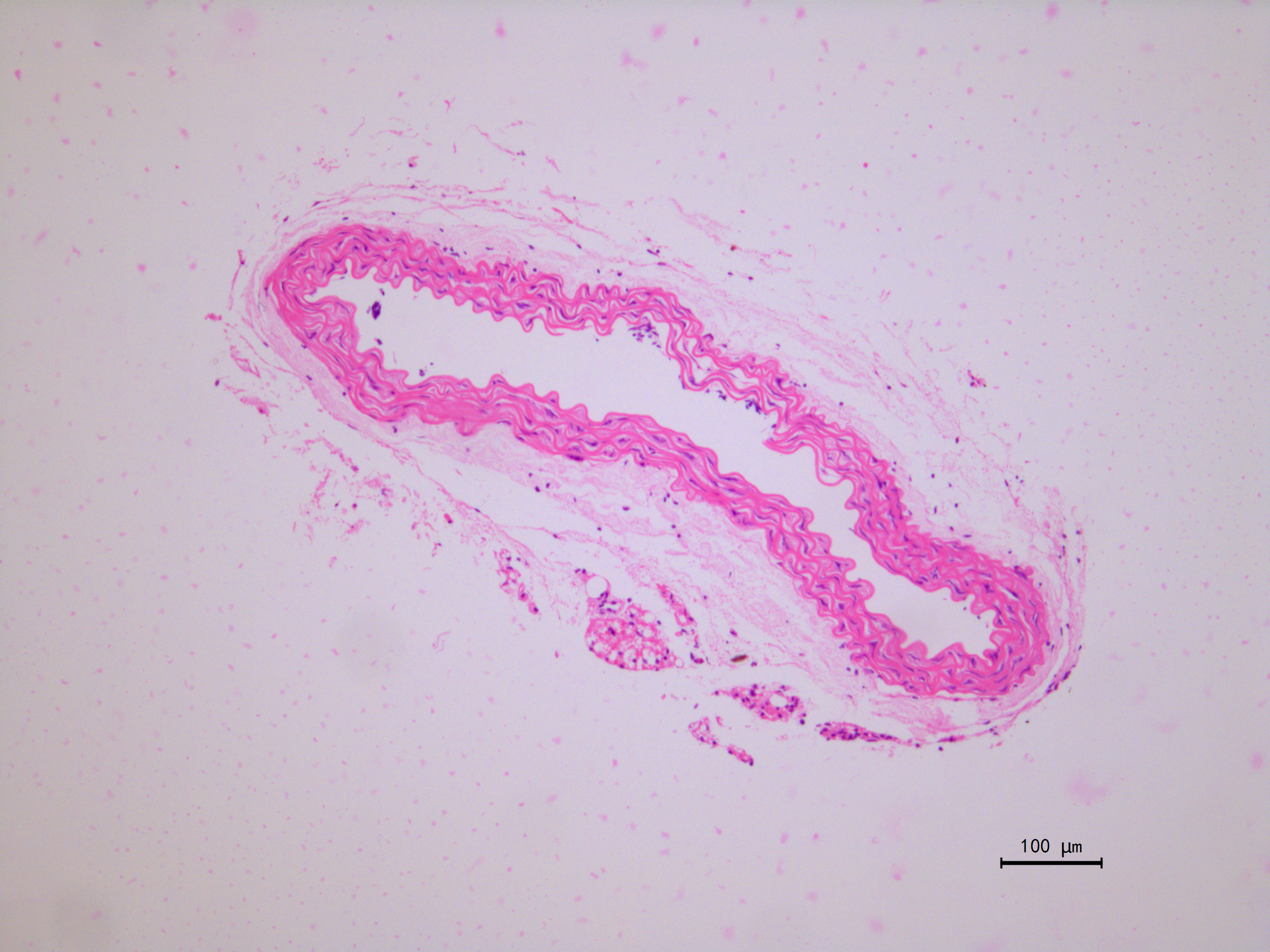

Supplement: S4 Fig — (TIF) [file pone.0314698.s005.tif]

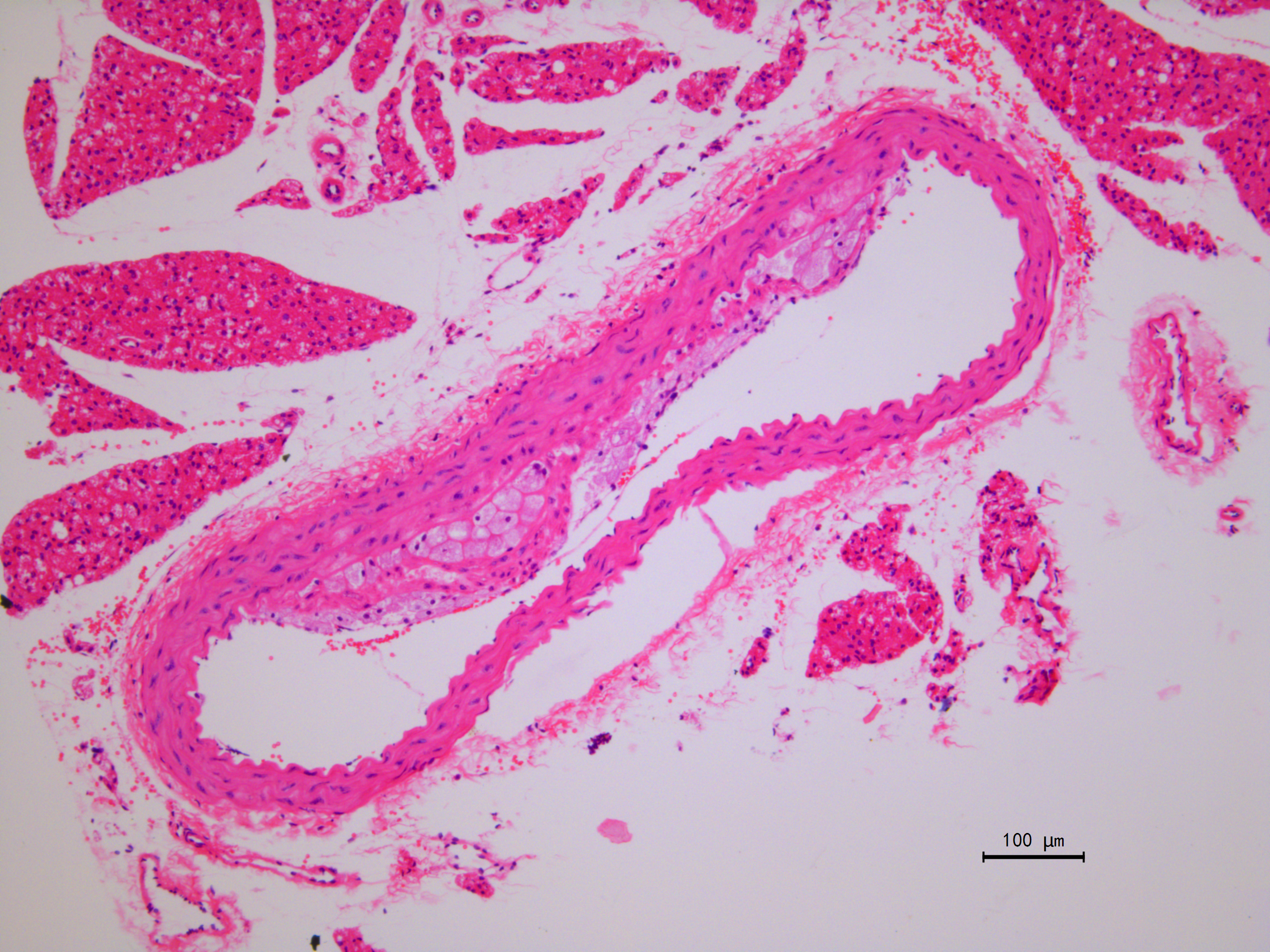

Supplement: S5 Fig — (TIF) [file pone.0314698.s006.tif]

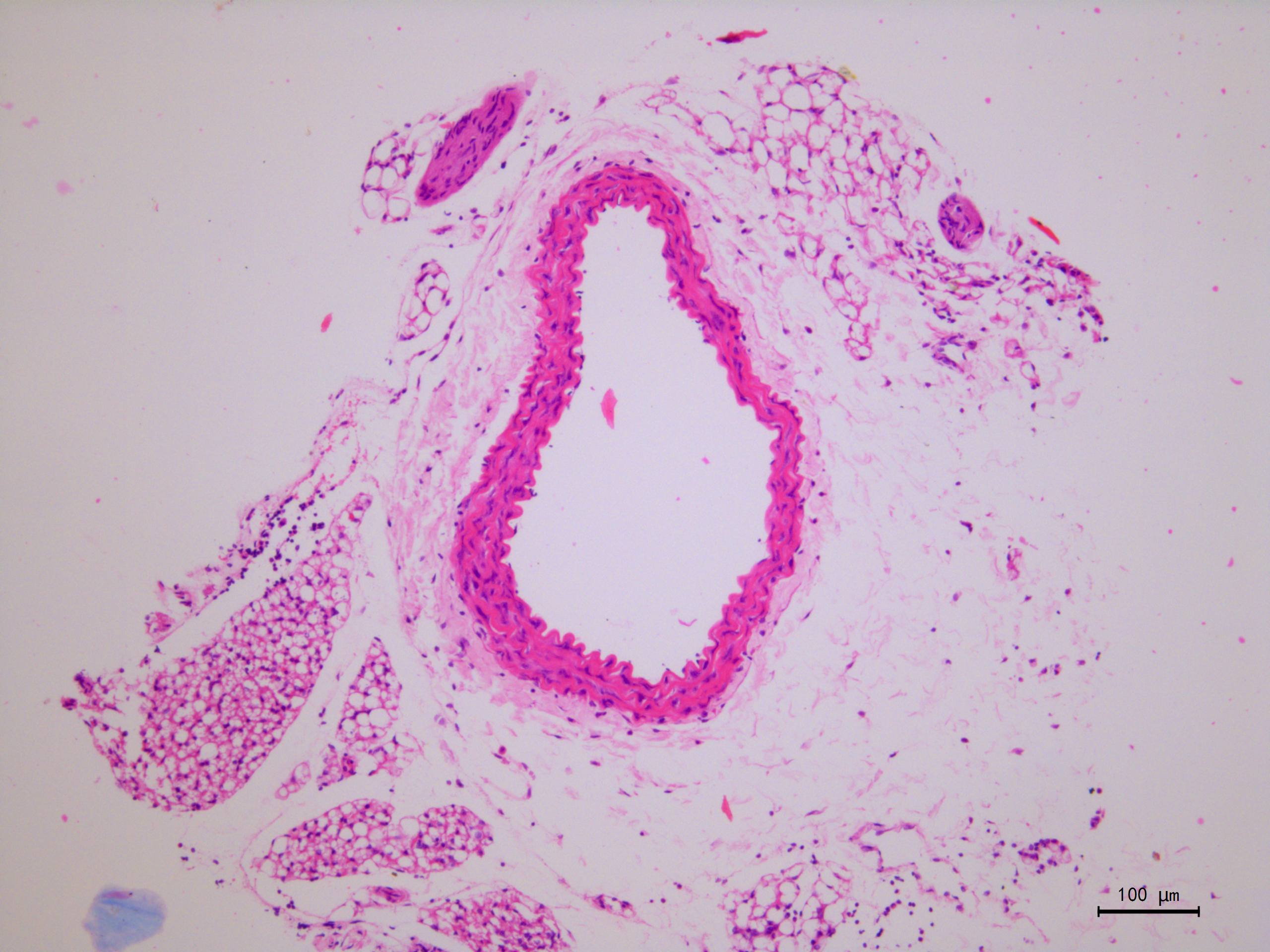

Supplement: S6 Fig — (TIF) [file pone.0314698.s007.tif]

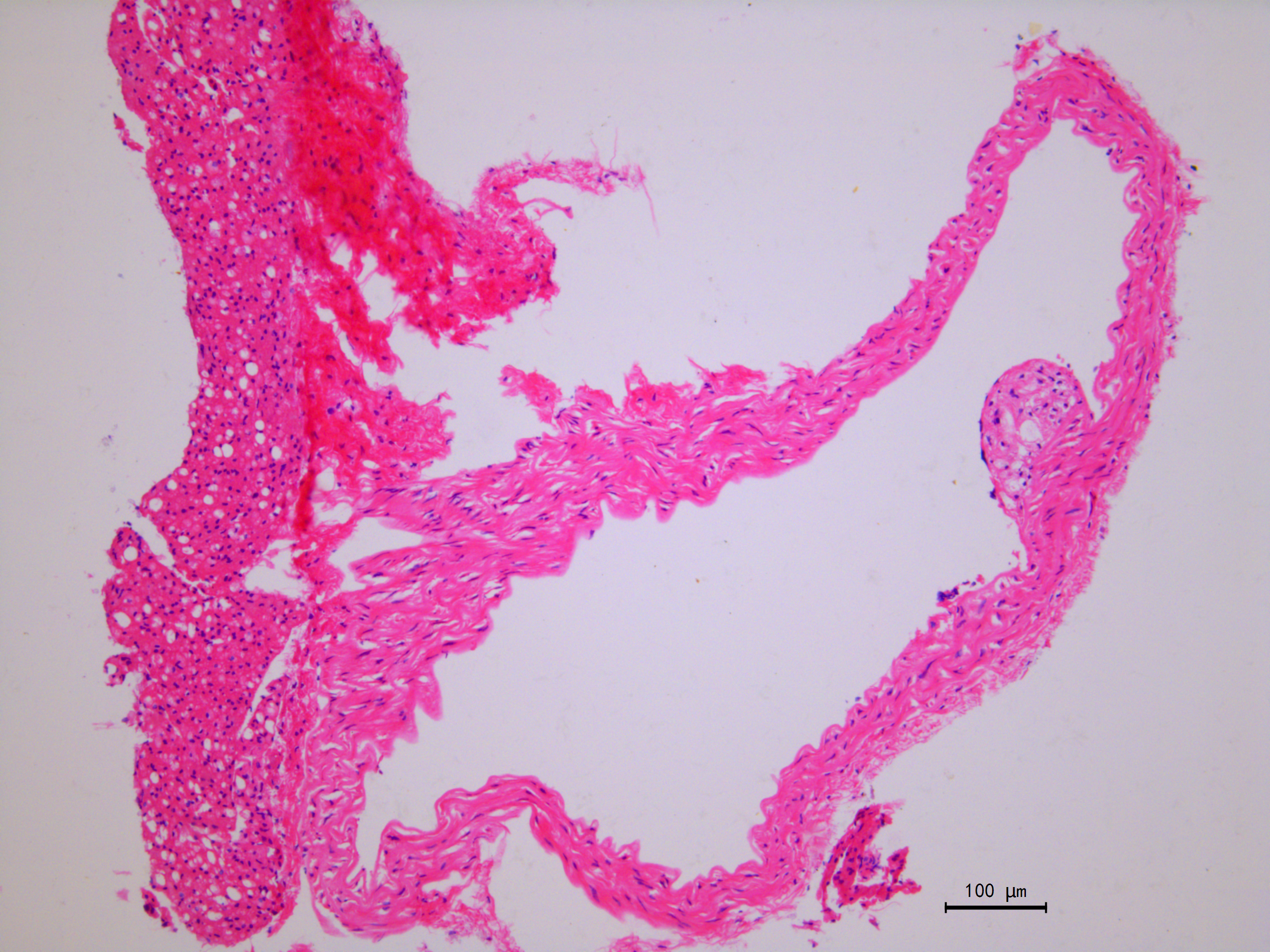

Supplement: S7 Fig — (TIF) [file pone.0314698.s008.tif]

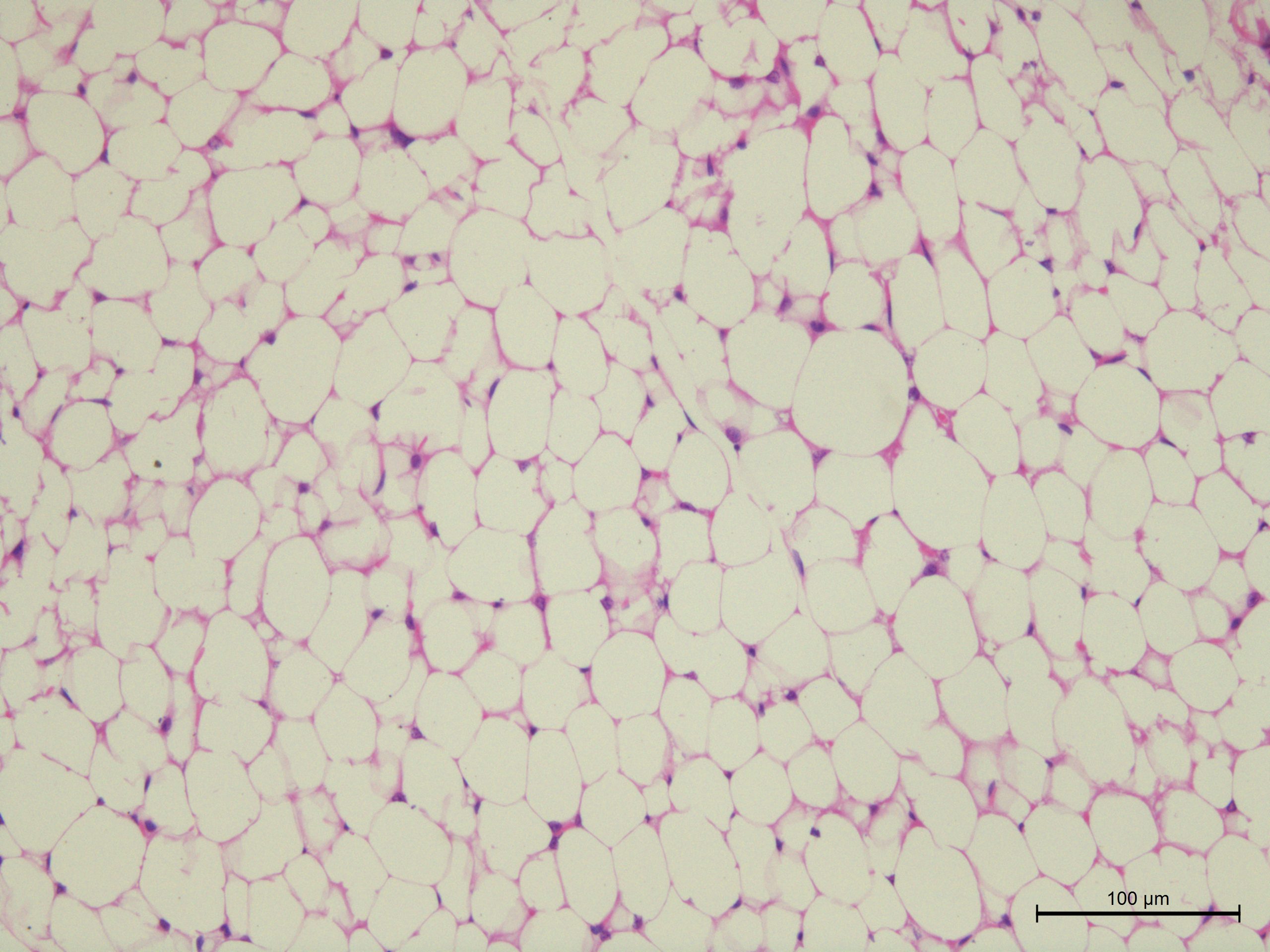

Supplement: S8 Fig — (TIF) [file pone.0314698.s009.tif]

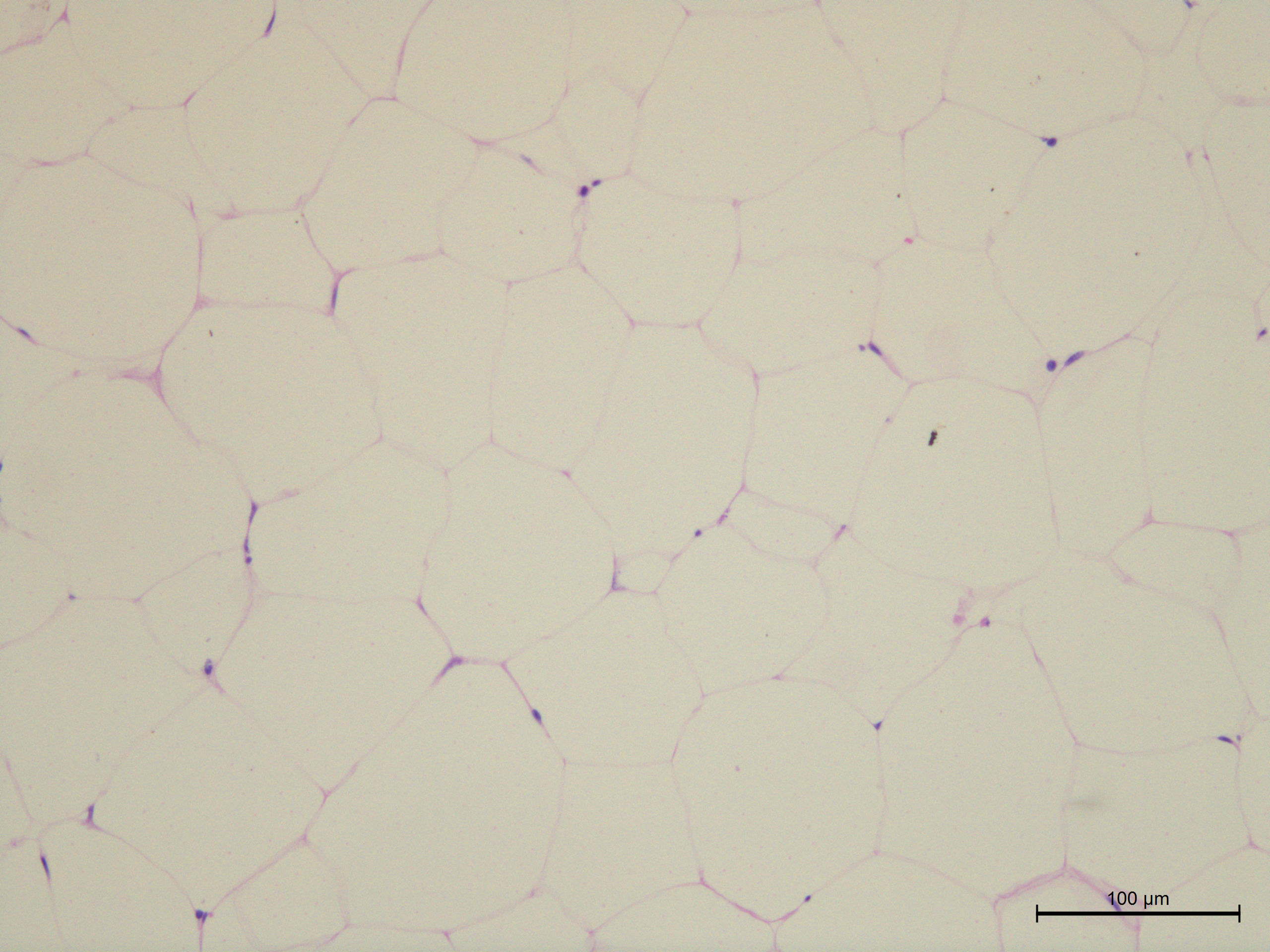

Supplement: S9 Fig — (TIF) [file pone.0314698.s010.tif]

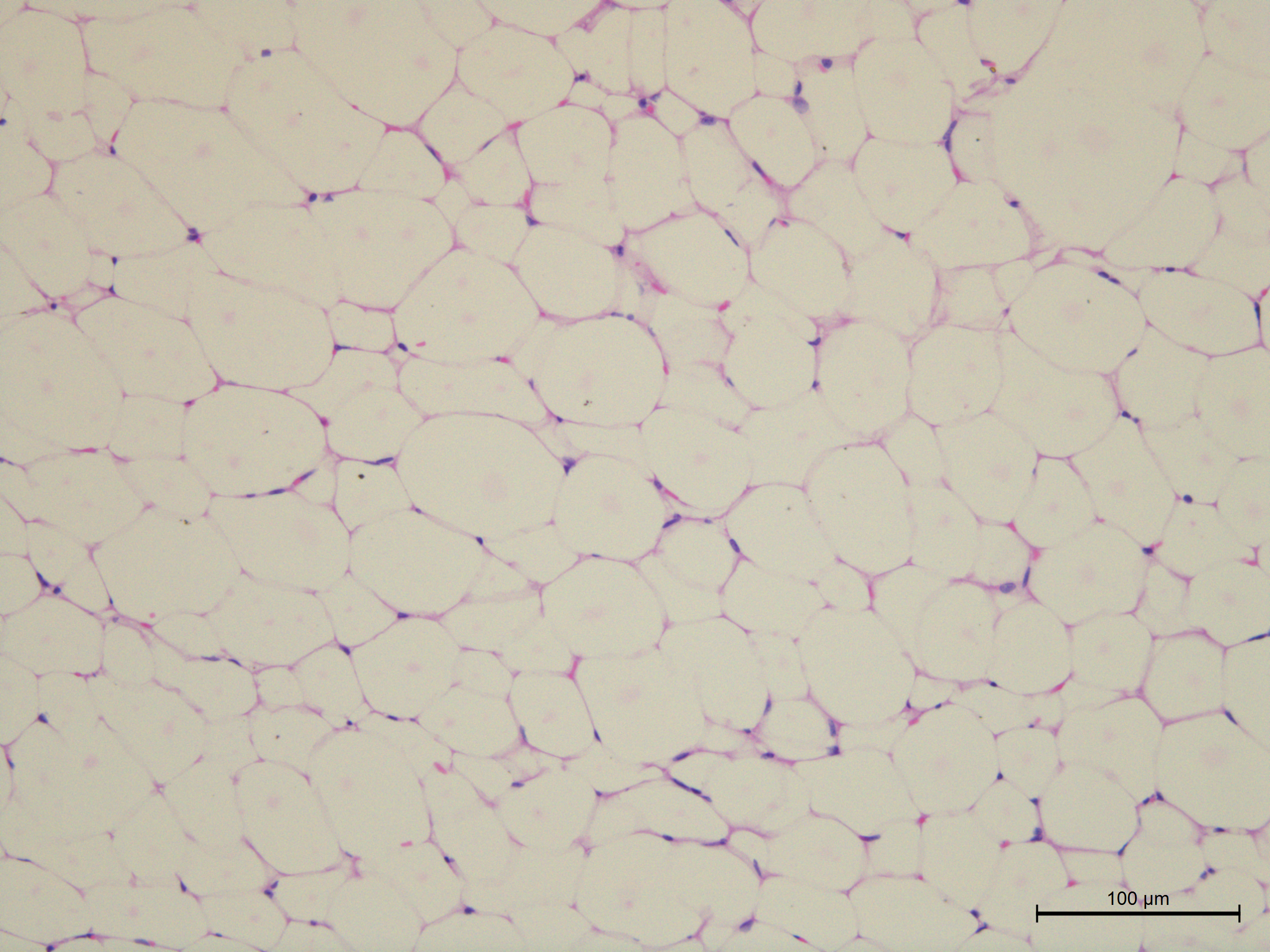

Supplement: S10 Fig — (TIF) [file pone.0314698.s011.tif]

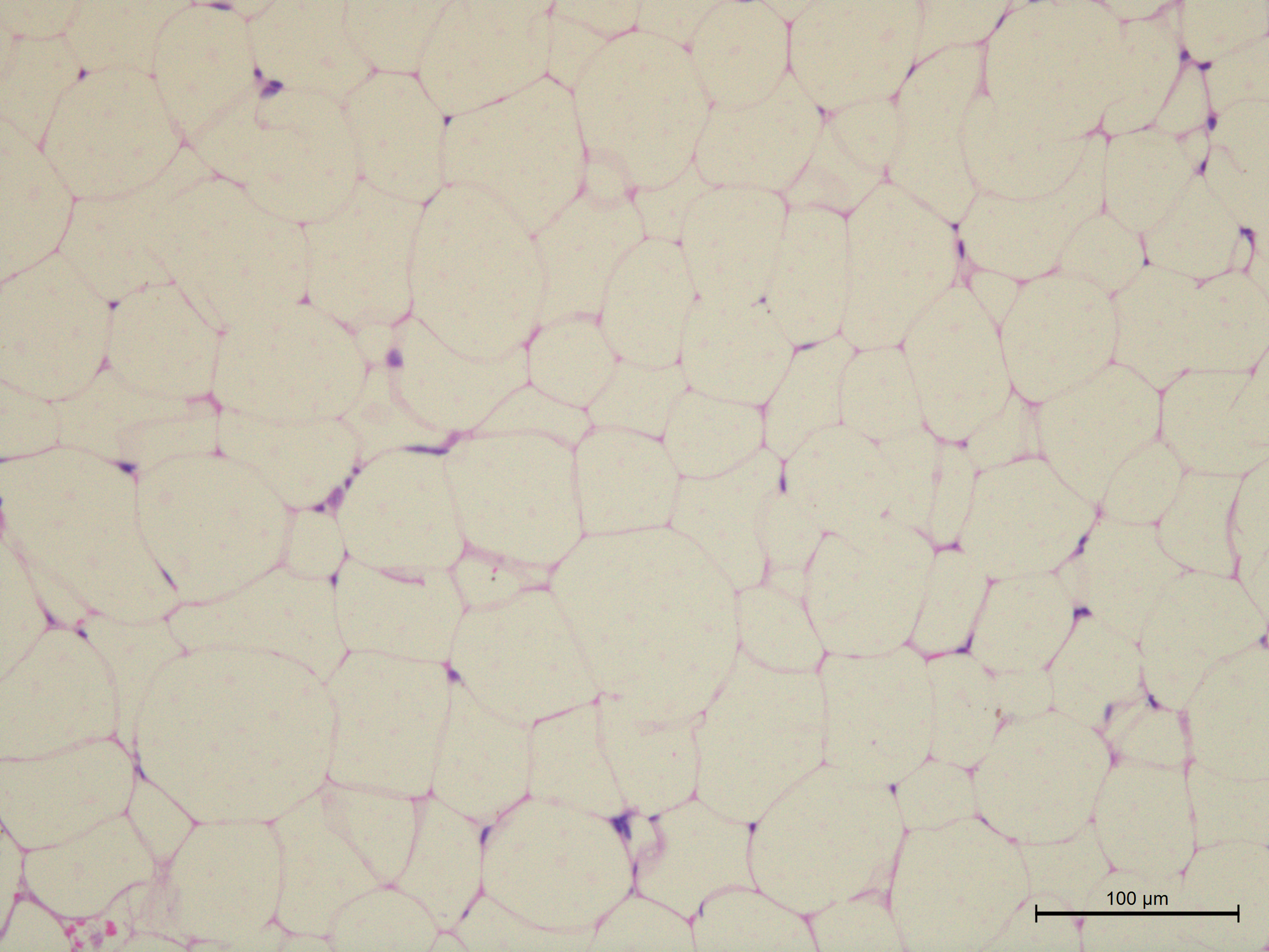

Supplement: S11 Fig — (TIF) [file pone.0314698.s012.tif]

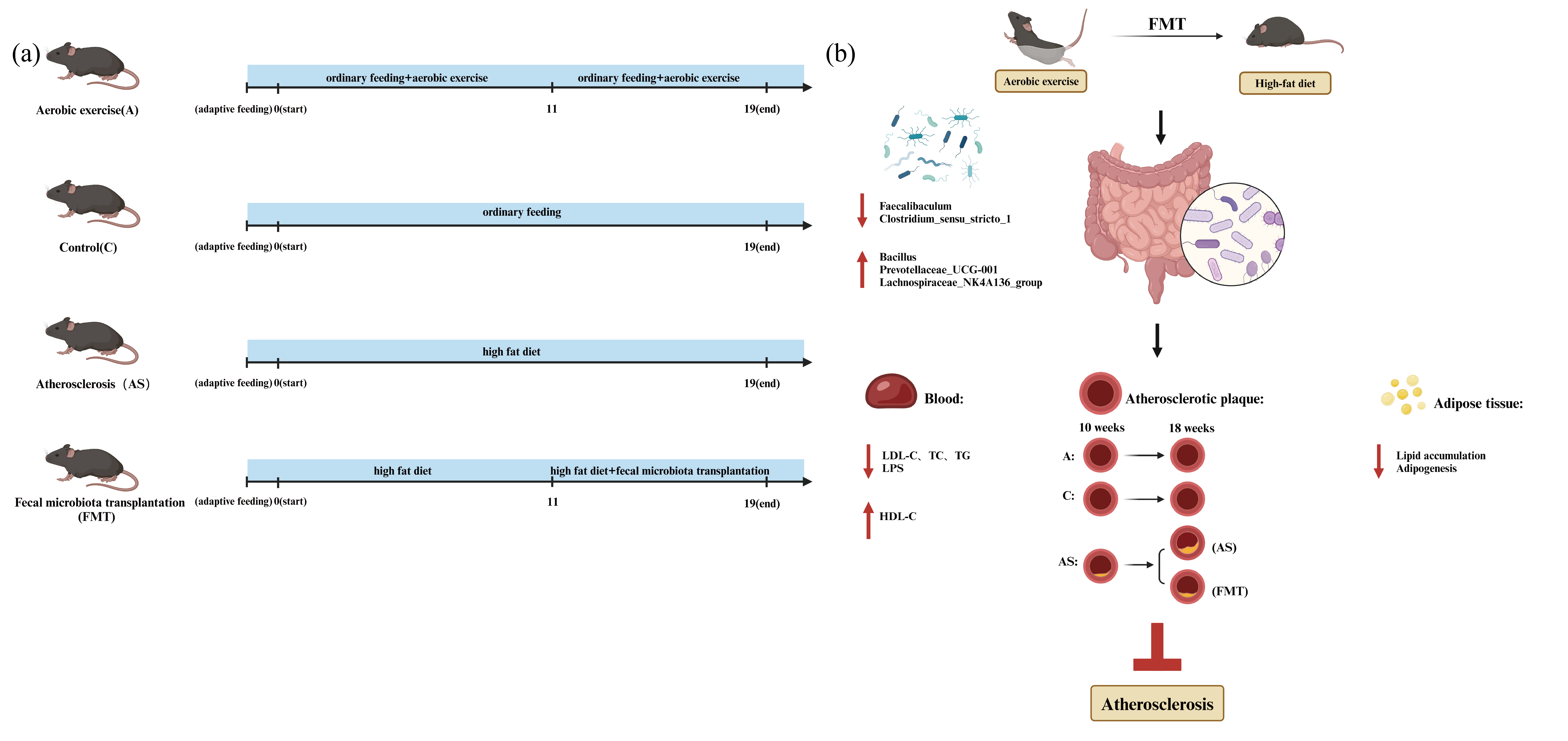

Supplement: S1 Graphical abstract — (PNG) [file pone.0314698.s013.png]
