# Supplementary material for: Fecal bacteria transplantation replicates aerobic exercise to reshape the gut microbiota in mice to inhibit high-fat diet-induced atherosclerosis
Source: PLoS One. 2025 Feb 4;20(2):e0314698. doi: 10.1371/journal.pone.0314698 (PMC11793757; doi:10.1371/journal.pone.0314698)
Supplement: Attachment — Submitted filename: Response to Reviewers.pdf [file pone.0314698.s014.pdf]

Response to Reviewers

Dear Editorial Board and Reviewers of PLOS ONE,

Hello! We sincerely appreciate your time in reviewing our manuscript and providing valuable feedback on our paper titled "Fecal bacteria transplantation replicates aerobic exercise to reshape the gut microbiota in mice to inhibit high-fat diet-induced atherosclerosis" (Manuscript Number: PONE-D-24-29677R1). Your insightful comments have greatly contributed to improving the current version, and we are truly grateful for the opportunity to revise our submission. The authors have carefully considered your suggestions and have provided detailed responses addressing the raised issues. We hope that the revised manuscript, with all modifications highlighted in red, meets your standards. It is our sincere hope that you will consider accepting this revised paper for publication in PLOS ONE. Please find our responses to your comments below:

Response to Reviewer 1

Comment #1: Authors are requested to reanalyze the statistical significance test for figure1e, figure 3b, c. Authors are suggested to prepare all the bar graphs with individual data points for better understanding. Authors are also necessary to check that data has been represented either mean ± standard error or mean ± standard deviation as they claimed mean ± standard error.

Response from the Authors:

Based on your valuable guidance and suggestions, our team has conducted an in-depth and meticulous review of the key data analyses in the manuscript, specifically addressing the statistical significance tests for the data presented in Figures 1e, 3b, and 3c. To ensure the accuracy and scientific rigor of the research results, we not only rechecked all related statistical processes but also conducted multiple verifications for potentially affected data sections, including a thorough review of the information presented in Figure 2.

During this process, we utilized GraphPad Prism 9.5 software tool - an industry-leading software - to comprehensively process, analyze, and graphically represent all relevant data. Ultimately, we have decided to express our data using the "mean ± standard deviation" format in Section 2.8 (Statistical Analysis) of the manuscript. This approach not only demonstrates our dedication to scientific rigor but also improves clarity in presenting research findings for peer reviewers and readers' accessibility. We are confident that these enhancements will further elevate both quality and credibility of this manuscript while appreciating your professional feedback that contributed towards refining and improving our work.

Comment #2: Authors have mentioned in material and methods section that they used Student's t-test and one-way ANOVA; otherwise, the Kruskal-Wallis. Authors are recommended to mention the tests used in figure 1 a, e; figure 2 and figure 3 b, c with number of samples/repeats (n =) in figure legends.

Response from the Authors:

We greatly appreciate your suggestion regarding the lack of detailed annotations of the specific statistical analysis methods in the experimental data figures. We sincerely apologize for this oversight in the initial draft and have taken immediate steps to correct and improve this aspect. After

thorough research and careful consideration, we confirm the following: Figure 1a was analyzed using Student's t-test; Figure 2 was analyzed using one-way ANOVA; and Figures 1e, 3b, and 3c were analyzed using the Kruskal-Wallis rank sum test, which is more appropriate for non-normally distributed data. Additionally, to enhance transparency and readability, we have precisely listed the sample sizes for each group in their respective figure legends, and we have also clearly indicated the specific statistical analysis methods used under each figure. These adjustments aim to ensure that our research report is more rigorous and standardized while demonstrating our commitment to academic integrity and high-quality scientific outcomes. Once again, we thank you for your insightful suggestions that undoubtedly helped us move towards higher standards.

Comment #3: Authors are suggested to add the significance test result in figure 1a as described result 3.1 section.

Response from the Authors:

We sincerely appreciate and value your insightful suggestions, which have prompted us to carefully optimize Figure 1a in order to enhance its clarity and readability. Specifically, we have now marked the time point at week 11 in the figure, clearly indicating that this marks the official start of fecal microbiota transplantation (FMT) intervention for the F group mice at the end of week 11. To further emphasize the statistical significance of the changing trend, we have added ** at week 12 based on detailed significance test results and *** between weeks 13 and 19 to reflect significant differences in body weight data between AS and F groups over time. These subtle yet meaningful adjustments not only enrich the information presented in the figure but also offer readers a more precise perspective to observe experiment progression and key findings. Once again, we appreciate your thoughtful guidance that has enlightened our path towards continuously refining our research and striving for the highest standards in every outcome.

Comment #4: Authors are recommended to add the lipids' name and LPS above the graphs of figure 2 to easy visualization for readers.

Response from the Authors:

We have labeled the corresponding lipid names or LPS above each legend in Figure 2 to improve its readability.

Comment #5: Authors are suggested to correct the excel data (supplementary) for Ace and Shannon index which is written oppositely.

Response from the Authors:

We sincerely apologize for misplacing the Ace and Shannon index data in Excel, which has now been corrected in the revised version. We greatly appreciate your scrutiny, and we will be more vigilant to prevent such oversights in the future, ensuring the maintenance of our research quality.

Comment #6: Authors are recommended to correct the appropriate reference mentioned as [16] in Introduction.

Response from the Authors:

We sincerely appreciate the profound insights you have provided. After carefully reviewing the references, we have made necessary changes in the revised version of the manuscript. Specifically, in the Introduction section, we have updated the reference content in [16] to:

“Considering the contraindications and risks of exercise, there are obstacles to promoting exercise therapy for AS among the elderly population [1]; Fecal microbiota transplantation (FMT) offers a novel approach for treating this condition.” Once again, we deeply appreciate your keen observation. Your feedback serves as an indispensable source of motivation for our continuous pursuit of excellence.

1. Aengevaeren VL, Mosterd A, Sharma S, Prakken NHJ, Möhlenkamp S, Thompson PD, et al. Exercise and Coronary Atherosclerosis: Observations, Explanations, Relevance, and Clinical Management. Circulation. 2020;141(16):1338-50. Epub 2020/04/21. doi: 10.1161/circulationaha.119.044467. PubMed PMID: 32310695; PubMed Central PMCID: PMCPMC7176353.

Response to Reviewer 2

Comment #1: Line 33, 209, 241, 303 and 343: Unknown figure legends with different front sizes. Need do necessary Changes.

Response from the Authors:

We greatly appreciate your meticulous feedback. In accordance with PLOS ONE guidelines, we have completely removed the blanks in #33, 209, 241, 303, and 343 from the revised manuscript. We sincerely apologize and thank you for bringing this to our attention.

Comment #2: Manuscript is pasted twice that need to be corrected and kindly use sample spacing format throughout the manuscript.

Response from the Authors:

Your insightful suggestions have enabled us to reflect and improve. I am pleased to inform you that we have thoroughly reviewed the revised manuscript in accordance with PLOS ONE's stylistic standards, making necessary adjustments to ensure full compliance with the journal's requirements. We are committed to presenting a more rigorous and professional version of our work. Your attention and guidance serve as the driving force behind our progress, for which we extend our deepest respect and gratitude.

Comment #3: Author must include any prebiotics, probiotics, and any standard weight loss medicine to compare the results, without that there is no positive control. Without that it is not rational to evaluate the study

Response from the Authors:

We deeply appreciate your professional insights and support for our research. Your suggestion to include a positive control group containing prebiotics, probiotics, and standardized weight management drugs has greatly enriched the scope of our study and reflects a forward-thinking approach. However, conducting such supplementary experiments at this stage presents significant technical and methodological challenges due to the following reasons:

The primary challenge arises from concerns over sample stability due to the long time span of the project. This research has been ongoing for two years, and although we have employed low-temperature storage (-80°C) to preserve the integrity of the mouse fecal samples, previous studies indicate that long-term freezing may lead to a decline in α-diversity, likely due to microbial DNA degradation[2]. Consequently, restarting the animal model would not only require replicating the initial conditions accurately to ensure consistency for the newly added control group but also

addressing uncertainties posed by variations in probiotic product efficacy, individual responses, and other external variables which could complicate comparability of experimental results.

Additionally, although an increasing body of evidence emphasizes that dysbiosis of gut microbiota (GM) is a potential contributor to atherosclerosis (AS), our understanding of its exact pathophysiological mechanisms remains limited [3]. While current research is gradually uncovering the functional interactions between GM and the host, the specific bacterial strains that should be introduced and the minimum effective dose required for intervention have not yet been clearly established[4]. A review of past studies[5, 6] shows that although changes in the microbiome and metabolites following FMT in AS model mice have demonstrated potential benefits, further systematic investigation is needed to comprehensively understand how these microbial components regulate the progression of AS as well as their distinct biological activities. Without a clear understanding of these underlying mechanisms, expanding the scope of FMT treatment could lead to unpredictable and difficult-to-interpret results.

Once again, we express our deep gratitude for your valuable feedback, which has significantly enhanced our study. We are committed to continuing our focus on investigating the molecular mechanisms through which exercise-mediated improvements in GM contribute to inhibiting AS. Our aim is to identify key bacterial strains and incorporate your suggestions into a more rigorous and refined experimental design. We firmly believe that by adhering to a rigorous, evidence-based scientific approach, we can unravel the complexities of biological phenomena and ultimately uncover the underlying causes of diseases, thereby contributing to human health and well-being.

2. Carroll IM, Ringel-Kulka T, Siddle JP, Klaenhammer TR, Ringel Y. Characterization of the fecal microbiota using high-throughput sequencing reveals a stable microbial community during storage. PLoS One. 2012;7(10):e46953. Epub 2012/10/17. doi: 10.1371/journal.pone.0046953. PubMed PMID: 23071673; PubMed Central PMCID: PMCPMC3465312.

3. Kim ES, Yoon BH, Lee SM, Choi M, Kim EH, Lee BW, et al. Fecal microbiota transplantation ameliorates atherosclerosis in mice with C1q/TNF-related protein 9 genetic deficiency. Exp Mol Med. 2022;54(2):103-14. Epub 2022/02/05. doi: 10.1038/s12276-022-00728-w. PubMed PMID: 35115674; PubMed Central PMCID: PMCPMC8894390.

4. de Groot PF, Frissen MN, de Clercq NC, Nieuwdorp M. Fecal microbiota transplantation in metabolic syndrome: History, present and future. Gut Microbes. 2017;8(3):253-67. Epub 2017/06/14. doi: 10.1080/19490976.2017.1293224. PubMed PMID: 28609252; PubMed Central PMCID: PMCPMC5479392.

5. Weingarden AR, Vaughn BP. Intestinal microbiota, fecal microbiota transplantation, and inflammatory bowel disease. Gut Microbes. 2017;8(3):238-52. Epub 2017/06/14. doi: 10.1080/19490976.2017.1290757. PubMed PMID: 28609251; PubMed Central PMCID: PMCPMC5479396.

6. Parker A, Romano S, Ansorge R, Aboelnour A, Le Gall G, Savva GM, et al. Fecal microbiota transfer between young and aged mice reverses hallmarks of the aging gut, eye, and brain. Microbiome. 2022;10(1):68. Epub 2022/05/04. doi: 10.1186/s40168-022-01243-w. PubMed PMID: 35501923; PubMed Central PMCID: PMCPMC9063061.

We sincerely appreciate the valuable feedback provided by the esteemed editorial board and reviewers on our manuscript. We eagerly anticipate your response

Thank you and best regards!
Yours sincerely,
Jie Men

Corresponding author:
Name: Jie Men
E-mail: menjie2020@126.com
